# Premature Vascular Aging in Guinea Pigs Affected by Fetal Growth Restriction

**DOI:** 10.3390/ijms20143474

**Published:** 2019-07-15

**Authors:** Adolfo A. Paz, German A. Arenas, Sebastián Castillo-Galán, Estefanía Peñaloza, Gabriela Cáceres-Rojas, José Suazo, Emilio A. Herrera, Bernardo J. Krause

**Affiliations:** 1 Department of Neonatology, Division of Paediatrics, Faculty of Medicine, Pontificia Universidad Católica de Chile, Marcoleta 391, Santiago 8330024, Santiago, Chile; 2 Programa de Fisiología, Facultad de Ciencias Biológicas, Pontificia Universidad Católica de Chile, Alameda 340, Santiago 8330024, Santiago, Chile; 3 Programa de Fisiopatología, Instituto de Ciencias Biomédicas, Facultad de Medicina, Universidad de Chile, Av. Salvador 486, Providencia 7500922, Santiago, Chile; 4 Instituto de Investigación en Ciencias Odontológicas, Facultad de Odontología, Universidad de Chile, Sergio Livingstone 943, Independencia 8380492, Santiago, Chile; 5 International Center for Andean Studies (INCAS), Universidad de Chile, Baquedano s/n, Putre, Chile

**Keywords:** Fetal Growth Restriction, Early Vascular Aging, Cardiovascular Diseases, Endothelial Dysfunction

## Abstract

Cardiovascular risk associated with fetal growth restriction (FGR) could result from an early impaired vascular function. However, whether this effect results in premature vascular aging has not been addressed. We studied the ex vivo reactivity of carotid and femoral arteries in fetal (near term), adults (eight months-old) and aged (16 months-old) guinea pigs in normal (control) and FGR offspring. Additionally, an epigenetic marker of vascular aging (i.e., LINE-1 DNA methylation) was evaluated in human umbilical artery endothelial cells (HUAEC) from control and FGR subjects. Control guinea pig arteries showed an increased contractile response (KCl-induced) and a progressive impairment of NO-mediated relaxing responses as animals get older. FGR was associated with an initial preserved carotid artery reactivity as well as a later significant impairment in NO-mediated responses. Femoral arteries from FGR fetuses showed an increased contractility but a decreased relaxing response compared with control fetuses, and both responses were impaired in FGR-adults. Finally, FGR-HUAEC showed decreased LINE-1 DNA methylation compared with control-HUAEC. These data suggest that the aging of vascular function occurs by changes in NO-mediated responses, with limited alterations in contractile capacity. Further, these effects are accelerated and imposed at early stages of development in subjects exposed to a suboptimal intrauterine environment.

## 1. Introduction

Cardiovascular disease (CVD) is the main cause of death worldwide [1]. Genetic changes associated with the traits of metabolic syndrome and cardiovascular diseases are able to explain a small proportion of risks and cases, suggesting the presence of other contributory factors in these conditions. Since Barker’s hypothesis of adult diseases’ origins during early life, the programming consequences in intrauterine life have been extensively studied [2]. Fetal growth restriction (FGR) includes any adverse condition that represses fetal growth potential, clinically defined in neonates whose birth weight is below the 10^th^ percentile for its gestational age along with signs of altered fetal or placental vascular function [3,4]. The mechanisms that could trigger FGR are vast, but a key factor is decreased placental perfusion, characterized by a reduction in the nutrients and oxygen supply to the developing fetus [5]. This fetal status has been related to morphological alterations in fetal arteries that favor a greater risk of cardiovascular diseases in adult life [6,7]. 

Compelling data have shown that FGR subjects have an adverse early vascular remodeling, which exhibits an increased aortic stiffness and a significantly higher aortic intima-media thickness; this has been proven in both humans and animal models, such as sheep and guinea pigs [8,9,10,11,12]. Nonetheless, whether or not these changes affect the contractile responses in lower- and upper-body arteries in a similar way, their immediate and long-term consequences remains elusive. It has been proposed that both structural and functional changes may occur in FGR, leading to early vascular aging (EVA) [13]. The latter is considered an unchronological and accelerated process of vascular senescence, characterized by endothelial dysfunction, an increased left-ventricular mass, and a higher systemic arterial pressure, all of which have been demonstrated to be permanent throughout adult life [14,15,16,17]. Conversely, FGR has been related to endothelial dysfunction programmed during fetal development that may persist during the life span. However, very few studies have addressed the effects of aging and FGR in the main traits associated with a healthy vascular reactivity. This study aimed to characterize life-course changes in vascular function and reactivity in normal guinea pigs. It then compared the immediate and long-term effects of fetal growth restriction in these responses. In order to provide further evidence of accelerated aging in pregnancies affected by an impaired fetal growth, the LINE-1 (Long interspersed nuclear element-1) DNA methylation level was evaluated in human umbilical artery endothelial cells derived from control and FGR subjects. 

## 2. Results

### 2.1. Life-Course Changes in the Ex Vivo Vascular Responses Control Guinea Pigs

**Figure 1:**
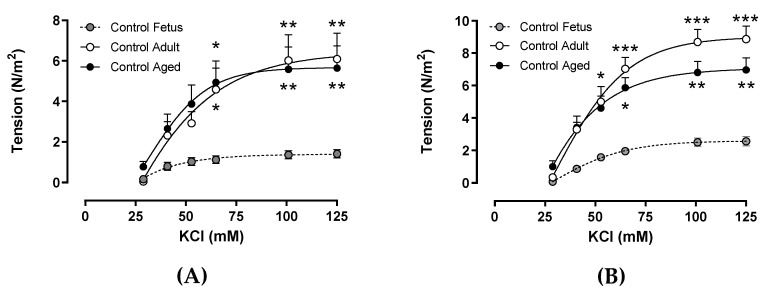
Ex vivo contractile response to KCl in arteries from fetal to aged guinea pigs. Concentration-dependent tension to KCl in carotid (**A**) and femoral arteries (**B**) from control fetus (gray circles, dashed black lines), control adult (open circles, continuous black lines), and control aged guinea pigs (solid circles, continuous black lines). Values are expressed in mean ± SEM. *p < 0.05, **p < 0.01, ***p < 0.001 vs term fetus. Two-way ANOVA.

Carotid arteries from control adult and control aged animals exhibited a comparable maximal constriction to increasing concentrations of KCl, and in both groups, their responses were significantly higher compared with the control fetuses (Figure 1A). Similarly, the maximal constriction in response to KCl in femoral arteries from the control aged and control adults was similar but higher than that of the control fetuses (Figure 1B).

**Figure 2:**
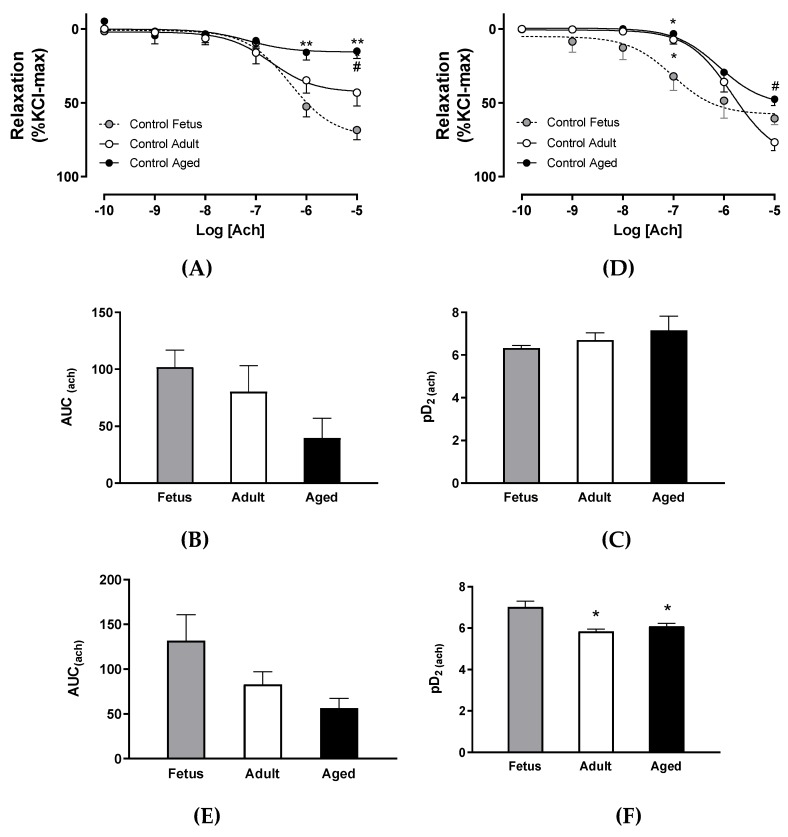
Ex vivo endothelial function in arteries from fetal to aged guinea pigs. Concentration-dependent relaxation in response to acetylcholine in carotid (**A**) and femoral arteries (**D**) from control fetus (gray circles, dashed black lines), control adult (open circles, continuous black lines), and control aged guinea pigs (solid circles, continuous black lines). Area under curve and pharmacological potency (pD_2_, i.e., sensitivity) of acetylcholine in carotid (**B**,**C,** respectively) and femoral arteries (**E**,**F,** respectively) from control fetus (gray bar), control adult (open bar), and control aged guinea pigs (black bar). Values are expressed in mean ± SEM. *p < 0.05, **p < 0.01 vs term fetus. #*p* < 0.05 vs adult. ANOVA.

Furthermore, carotid arteries from control aged animals exhibited a lower maximal relaxation in response to acetylcholine compared with control adults and fetuses (Figure 2A) without changes in total relaxation (AUC) (Figure 2B) and potency (Figure 2C). In addition, femoral arteries from aged guinea pigs showed a lower acetylcholine-induced relaxation compared to control adults (Figure 2D), while potency was decreased in adult and aged animals (Figure 2F) without differences in the total relaxation among groups (Figure 2E). 

**Figure 3:**
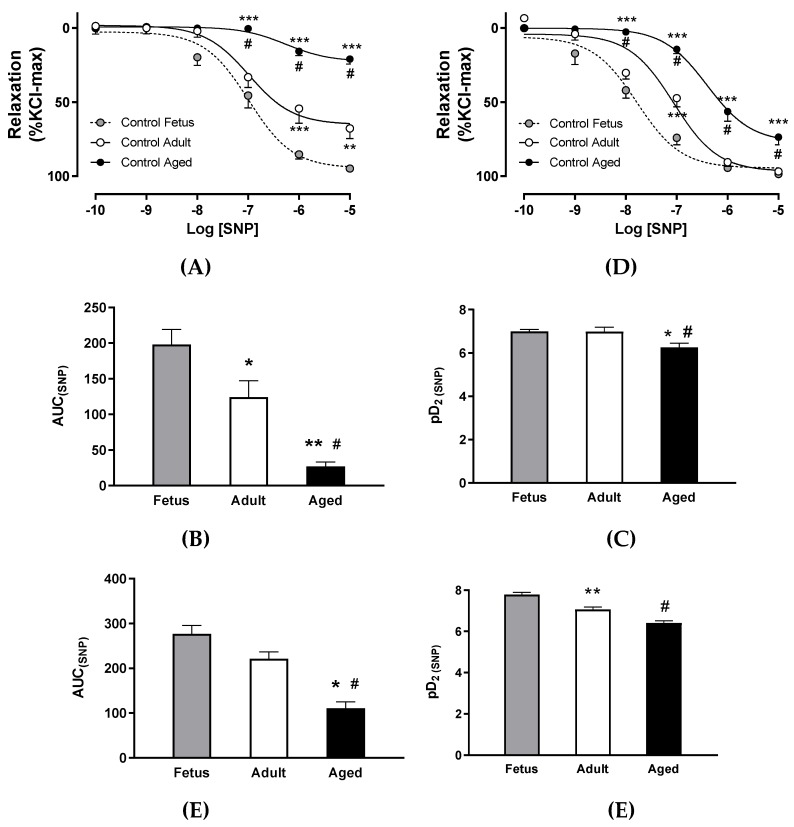
Ex vivo NO-dependent relaxation in arteries from fetal to aged guinea pigs. Concentration-dependent relaxation in response to sodium nitroprusside (SNP) in carotid (**A**) and femoral arteries (**D**) from control fetus (gray circles, dashed black lines), control adult (open circles, continuous black lines), and control aged guinea pigs (solid circles, continuous black lines). Area under curve and pharmacological potency (pD_2_, i.e., sensitivity) of acetylcholine in carotid (**B**,**C**, respectively) and femoral arteries (**E**,**F**) from control fetus (gray bar), control adult (open bar), and control aged guinea pigs (black bar). Values are expressed in mean ± SEM.*p< 0.05, **p < 0.01, ***p < 0.001 vs term fetus. #*p* < 0.05 vs adult. ANOVA.

Life-course changes in endothelial-independent relaxation were analyzed using sodium nitroprusside (SNP), an NO donor. Carotid arteries from control adults showed a decreased maximal (Figure 3A) and total relaxation (Figure 3B) in response to SNP compared with control fetuses, and this was further impaired in aged animals. SNP potency was comparable between carotid arteries from control fetuses and adults, but it was reduced in aged animals (Figure 3C). In contrast, femoral arteries from control fetuses and adults showed a comparable maximal response (Figure 3D) and total relaxation (Figure 3E), and these parameters were decreased in aged animals. SNP potency was decreased in control adults compared with control fetuses—an effect that was further affected in control aged (Figure 3F).

### 2.2. Effect of FGR on Offspring Morphometry

At the term of gestation, FGR animals presented lower body, heart and kidney weights compared with control fetuses. However, relative heart and kidney weights were comparable between groups (Table 1). Conversely, adult FGR guinea pigs showed a decrease in kidney weight and relative kidney weight compared with control adults, with no differences in body and heart weight.

### 2.3. Effect of Early Growth Restriction on the Vascular Responses of Fetal and Adult Guinea Pigs

**Figure 4:**
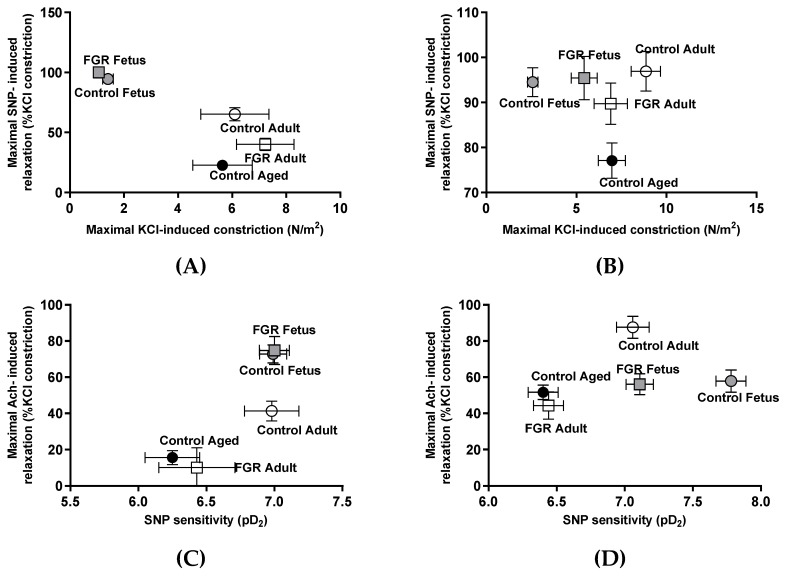
Effects of fetal growth restriction (FGR) in life-course changes in the ex vivo contractile and relaxing responses in guinea pig carotid and femoral arteries. Maximal contractile capacity (KCl-induced constriction) and maximal relaxation to exogenous NO in carotid (**A**) and femoral (**B**) arteries from control and FGR fetus and adults, as well as control aged guinea pigs. Sensitivity to exogenous NO (pD_2_) and maximal relaxation response to acetylcholine in carotid (**C**) and femoral (**D**) arteries from control and FGR fetus and adults, as well as control aged guinea pigs. Values derived from Table 2.

The values of internal diameter, as well as maximal responses to KCl, acetylcholine, SNP, and SNP potency in carotid and femoral arteries from control and FGR guinea pigs are reported in Table 2. FGR was associated with a decreased internal diameter in the carotid artery at the end of gestation compared with control fetuses, but this effect was not observed in adult FGR subjects. In order to integrate the lifelong effects of FGR on carotid and femoral artery ex vivo responses, the dynamic contractile and relaxing ranges (i.e., maximal KCl and SNP responses), as well as the interaction between endothelial-dependent relaxation and the sensitivity to NO in the smooth muscle layer (i.e., maximal acetylcholine response and SNP potency), were compared. Maximal contractile and relaxing responses showed that carotid arteries from FGR fetuses had a comparable reactivity to control fetuses. Furthermore, the major changes observed in FGR adults and aged controls were related with a decreased endothelial-independent relaxing response compared with control adults (Figure 4A). In contrast, femoral arteries from FGR fetuses and adult animals, either control or FGR, showed a comparable endothelial-independent relaxing response and an increased contractile tension compared with control fetuses (Figure 4B).

Conversely, the integration of sensitivity to NO (i.e., SNP potency) and maximal endothelial-dependent relaxation (EDR) showed comparable responses between control and FGR fetal carotid arteries. In addition, the main changes in control adults were related to a decrease in maximal EDR that was further impaired in control aged and FGR adults (Figure 4C). In contrast, femoral arteries from FGR fetuses showed a lower SNP potency compared with control fetuses, thought this potency was comparable to control adults. Meanwhile, FGR adults had a lower sensitivity compared with control adults, though this sensitivity was similar to control aged animals (Figure 4D). 

### 2.4. LINE-1 DNA Methylation in HUAEC

**Figure 5:**
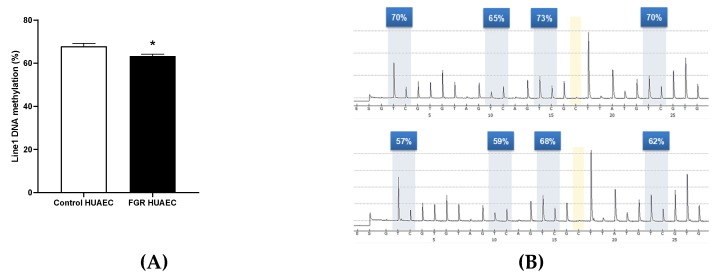
Epigenetic marks of aging in control and FGR human umbilical artery endothelial cells. Average DNA methylation levels (**A**) and representative programs (**B**) of methylated CpG sites from LINE-1 gene in control (open bar, right upper panel) and FGR (solid bar, right lower panel) human umbilical artery endothelial cells (HUAEC). Values are expressed in mean ± SEM.*p < 0.05.

In order to determine whether or not FGR in humans results in an epigenetic signature of vascular aging, LINE-1 DNA methylation was analyzed in human umbilical artery endothelial cells (HUAEC) from control and FGR pregnancies. The average (Figure 5A) and CpG-specific (Figure 5B) LINE-1 DNA methylation was lower in FGR HUAEC compared with control cells.

## 3. Discussion

This study aimed to characterize life-course changes in the general markers of vascular reactivity in control guinea pigs and to compare the immediate and long-term effects of fetal growth restriction in these responses. Furthermore, we studied vascular early aging in umbilical cords from control and FGR pregnancies in women. 

By studying the ex vivo responses of carotid and femoral arteries from term fetuses, adult, and aged guinea pigs, it was found that major changes in vascular reactivity were related with an increased contractile response between fetal and adult/aged subjects and the progressive impairment of vasodilator function. Conversely, we integrated the effects of altered fetal growth on the contractile-relaxing dynamic range and the vascular reactivity of term fetuses and adults FGR. FGR was associated with a preserved fetal carotid artery reactivity; however, at adult age, there was a significant impairment in NO-mediated vascular responses, comparable to aged animals. In contrast, femoral arteries from FGR fetuses showed a reactivity, in terms of the contractile-relaxing dynamic range and NO-mediated responses, comparable to control adults that were further impaired at adult age. Finally, arterial endothelial cells from human FGR pregnancies showed an epigenetic signature associated with vascular aging. Altogether, these data suggest that vascular function aging occurs mainly by changes in NO-mediated responses with limited alterations in contractile capacity, and this effect is accelerated and imposed at the early stages of development in subjects exposed to a suboptimal intrauterine environment. 

Aging has been related to a progressive impairment of vascular function resulting from an increased contractile capacity and a reduction in NO-mediated responses. However, the contribution of each mechanism has not been completely defined. A recent study in humans showed that subcutaneous small arteries exhibit a progressive pro-constrictive remodeling with aging and in hypertensive subjects [18]. In order to gain insights of this process, we studied contractile responses using a non-mediated constrictive agent (KCl) whose responses are directly related with the biomechanical and structural characteristic of a vessel [10]. Here, we found that adult guinea pig arteries had an increased contractile response to KCl, which is directly related with the relative presence of smooth muscle cell in the vascular wall, compared with term fetuses; however, this response was not further increased in aged animals. Other reports have shown dissimilar consequences of aging on the contractile responses in isolated vessels. For instance, an increased contractile response to KCl among young, adult, and aged rats has been found in carotid but not in basilar arteries [19]. In contrast, femoral arteries from adult rats have been reported with a preserved [20] or increased [21] contractile response to adrenergic agents. These controversies have also been observed in mesenteric arteries from mice [22] and rats [23,24,25]. Altogether, these data suggest that the contribution of contractile responses to vascular aging remains unsolved, and further studies are required to determine the potential species- and arterial bed-specific changes occurring in this process.

Furthermore, endothelial function and the NO vasodilator pathway have been suggested to play a central role in the progression of vascular aging. Studies in humans have shown that flow-mediated dilation, as a proxy of endothelial-dependent vasodilation [26,27], is impaired in aged adults compared with young adults [28,29,30,31]. These effects are enhanced by a sedentary life-style [29,31]. Studies of vascular aging in rats have shown a progressive but subtle impairment in the endothelial function in the aorta and mesenteric arteries. The latter results mainly from a decreased sensitivity to vasorelaxing agents, with no changes in the responses to exogenous NO [32,33,34]. However, other studies have shown no conclusive effects of aging on endothelial-dependent relaxation in femoral [20,21] or mesenteric [23,35] arteries. Here, we found that aging was associated with an arterial-bed specific impairment on NO-mediated vasodilation. In fact, carotid arteries showed a progressive reduction in the endothelial-dependent and independent responses to NO, mainly due to a decrease in maximal responses. In contrast, the impaired NO-mediated relaxation in femoral arteries in adult and aged subjects resulted from a decreased sensitivity to vasodilator agents. A recent report studying the ex vivo vascular reactivity in human subcutaneous small arteries showed that NOS-dependent vasodilation is progressively decreased with aging [18]. Contrariwise, several studies in humans [28,29,30,31] and animals [20,21,23,32,33,34] have suggested that vascular aging occurs with no changes in response to NO-donors. Nonetheless, a metanalysis of vascular function reports in humans has suggested that aging is associated with an impaired NO-induced response, which is evident mainly in large vessels compared with small arteries [36]. Altogether, these data suggest that the impaired NO-mediated vasodilation occurring in aging could result from a combined contribution of endothelial and vascular smooth muscle dysfunctions which are arterial bed-specific. 

Compelling evidence has shown that impaired fetal growth could be a significant determinant of vascular risk at long-term [37]. In fact, a comparison of the odds ratio for hypertension, suggested that the best-characterized gene polymorphisms is far lower [38] than those attributed to an altered fetal growth [39]. Conversely, as has been previously discussed, the aging-related changes in vascular function may result from a combination of increasing contractile responses and impaired vasodilator effects. In order to integrate the lifelong effects of FGR on the aging of vascular function, the dynamic contractile and relaxing ranges (characterized in this study by the maximal KCl and SNP responses), as well as the interaction between endothelial-dependent relaxation and the sensitivity to NO in the smooth muscle layer, were compared. The contractile and relaxing responses in FGR guinea pig arteries, both carotid and femoral, followed a pattern of accelerated aging, an effect that was evident since late gestation in femoral arteries but not in carotid vessels. Notably, the changes in NO-mediated responses occurred parallel to life-course modifications in eNOS expression and activation [40,41]. Comparable studies in fetal growth-restricted rats have shown sex-specific early aging in the vascular responses of mesenteric arteries, occurring in females but not in male offspring [23,35]. However, a further analysis of these studies shows that flow-mediated vasodilation was similarly impaired in young and aged FGR rats, independently of sex [23]. Our limited number of animals studied in adult and aged groups did not allow us to determine potential sex-specific effects. Nonetheless, vascular dysfunction in adult FGR guinea pigs, either male or female, has been previously shown [42].

In order to complements our observations, we determined whether an impaired fetal growth might result in early molecular markers of vascular aging in human endothelial cells. To address this, we determined the presence of an epigenetic marker of aging (i.e., LINE-1) in human endothelial cells from FGR subjects. Previous reports have shown that FGR, in human and animal models, results in comparable epigenetic changes in the endothelium. For instance, a similar epigenetic effects occurs in the gene coding for eNOS (*NOS3 or Nos3*) in human [43,44], guinea pig [40,41], and rat [45] FGR endothelial cells. Conversely, LINE-1 has been suggested to correlate with aging, cardiovascular risk, and metabolic dysfunction in humans [46,47,48,49,50], conditions prompted by an impaired fetal growth. Our results showed that FGR in humans occurred with an epigenetic signature in LINE-1 associated with vascular aging and dysfunction [51]. Previous studies in placental tissue from subjects with an impaired fetal growth showed no changes in the LINE-1 DNA methylation level compared with controls [52,53]. However, a study based on a large cohort of mother-child dyads (*n* = 319) showed that LINE-1 methylation in cord blood samples but not placental tissue is associated with an impaired fetal growth [54]. This stronger correlation between impaired fetal growth and LINE-1 DNA methylation in fetal tissues relative to placenta has been reported in a rat model of FGR [55]. Altogether, these data suggest that epigenetic changes resulting from FGR may be comparable among diverse mammals. In this context, here we provide further evidence, by studying a single cell type, that FGR results in epigenetic markers in the endothelium that would be also associated with accelerated aging. However, whether these markers remain at long-term need to be elucidated [41].

At our best knowledge, our study addressed, for the first time, the changes in vascular reactivity in guinea pigs at different stages of life, as well as the consequences of impaired fetal growth. In summary, our results suggest that vascular aging results from a concomitant endothelial and vascular smooth muscle dysfunction that occurs in an arterial bed-specific manner. In this context, fetal growth restriction leads to premature vascular aging, evidenced by early epigenetic markers in the endothelium and an impaired endothelial function in femoral arteries.

## 4. Materials and Methods

### 4.1. Ethics Statement

All animal care, procedures, and experimentation were approved by the Bioethics Committee of the Faculty of Medicine, Universidad de Chile (protocol CBA# 0694 FMUCH, March 2018) and were conducted in accordance with the ARRIVE guidelines and the Guide for the Care and Use of Laboratory Animals published by the US National Institutes of Health (NIH Publication No. 85–23, revised 1996). In addition, this study was performed with the ethical approval of the Ethics Committee from the Faculty of Medicine, Pontificia Universidad Católica de Chile (1130801 & 1181341), according to the principles outlined in the Declaration of Helsinki for the use of human tissue. 

### 4.2. Human Umbilical Cord and Placental Samples

Participants provided written informed consent before the obtention of their placenta and umbilical cord samples. Placentae were collected after delivery from full-term normal (control group) and FGR-diagnosed pregnancies from normotensive, non-smoking, non-alcohol, or drug consuming mothers, without intrauterine infection or any other medical or obstetrical complication. Gestational age estimated by ultrasonography before the 12th week of pregnancy was considered; human FGR was defined as fetal body weight below the tenth percentile adjusted for gestational age and sex, along with a low abdominal circumference or an altered Doppler registry, as previously reported [56]. 

### 4.3. Animals & Experimental Design

Twenty-one adult female Pirbright White guinea pigs (*Cavia porcellus*) were used for this study. All animals were housed in individual cages under standard conditions (30–35% humidity, 21–22 °C, and a 12: 12-h light-dark cycle), with a controlled food-by-body weight intake with a commercial diet (LabDiet 5025, Guinea Pigs, 20–30 g/d). After confirming pregnancy, the pregnant guinea pigs were randomly assigned to the control (*n* = 12) or FGR (*n* = 9) groups. All pregnant sows were subjected to aseptic surgery under general anesthesia on gestation day 35, either sham-operated (control) or to progressive uterine artery occlusion (FGR) [12,40].

### 4.4. Euthanasia

At 60–62 days of gestation (~90% of pregnancy), control (*n* = 12) and FGR (*n* = 12) fetuses were euthanized with a maternal anesthetic overdose (sodium thiopental 150 mg/kg IP, Opet, Laboratorio Chile). Conversely, at 8 (6 control adult and 6 FGR adult) and at 6 (6 control aged) months-old, the animals were euthanized with an anesthetic overdose (Sodium Thiopentone 150 mg/kg IP, Opet, Laboratorio Chile). Once the cardio-respiratory arrest was confirmed, the femoral, carotid, and aortic arteries were carefully obtained and clean.

### 4.5. Carotid and Femoral Arteries Vascular Function

Carotid and femoral arteries were excised, and 2 mm segments were mounted on a wire myograph (610M System Myograph multiwire, DMT) to determine vasoactive responses as previously reported [40]. The internal diameter for each vessel sample was defined by determining the maximal stretch-to-contractile relationship, a method that allowed us to reach a resting tone representative of in vivo conditions [57,58,59]. Contractile responses to increasing concentrations of KCl (25–125 mM) were determined as a proxy of the biomechanical and functional properties, and tension was reported as the force relative to the vessel area (N/m^2^) [10]. In order to determine vasodilator responses, vessels were pre-constricted with a half-maximal KCl concentration (40.8 mmol/L). The NOS-dependent vasodilation was assessed as the response to cumulative concentrations of acetylcholine (10^−8^–10^−5^ mol/L) and reported as the difference between the response in the absence and presence of an NOS inhibitor (L-NAME, 100 µmol/L). The NOS-independent response to NO was determined with sodium nitroprusside (SNP, 10^−9^–10^−5^ mol/L) in pre-constricted vessels [40].

### 4.6. Human Umbilical Artery Endothelial Cell

Human umbilical artery endothelial cells (HUAEC) were isolated by collagenase digestion from the placentae and umbilical cords of control and FGR pregnancies. Cells were cultured in medium 131 with MVGS (Microvascular Growth Supplement, S00525, Invitrogen Waltham, Massachusetts, USA) up to the second passage and then starved (2% serum) overnight prior to the extraction of DNA as previously described [56]

### 4.7. LINE-1 DNA Methylation

The methylation status of the LINE-1 was determined using a bisulfite modification coupled to DNA sequencing [56]. Briefly, total DNA extracts from HUAEC were treated with sodium bisulfite to convert unmethylated cytosine to uracil, and promoter regions were amplified by PCR using specific primers for LINE-1 reported elsewhere [60,61,62]. One of the PCR primers was designed to include a 5’-biotin tag, which allowed for the purification of the amplicon directly from the PCR mix. Then, a biotin-containing amplified DNA strand was transferred to a PyroMark Q96 MD pyrosequencer (Qiagen, Basel, Switzerland) for sequencing. CpG methylation was determined as the percentage in the four CpG sites present in LINE-1, comparing the signal intensity of non-bisulfite-sensitive CpG and bisulfite-sensitive CpG.

### 4.8. Data and Statistical Analyses

Data were expressed as mean ± SEM. Concentration-response curves were analyzed using an agonist-response best-fit line, where the maximal vasomotor response was expressed as a percentage of the contraction induced by 40.8 mM K^+^ (%Kmax for relaxation), and the vascular sensitivity was expressed as pD2 (-logEC_50_) [40,56,63]. The DNA methylation levels in HUAEC were compared with a non-parametric *t* test. Differences were considered significant when *p* ≤ 0.05 (Prism 5.0; GraphPad Software). The specific statistical analysis for each finding is indicated in figure footnotes.

## Figures and Tables

**Table 1:** Fetal, adult, and aged body, heart, and kidney weights.

Fetuses	Control (*n* = 12)	FGR (*n* = 12)	
Body (g)	82.5 ± 3.0	53.8 ± 7.1**	
Heart (g)	0.58 ± 0.02	0.46 ± 0.04*	
Kidney (g)	0.76 ± 0.02	0.61 ± 0.05**	
Rel. heart weight	0.71 ± 0.02	0.71 ± 0.03	
Adults	Control (*n* = 6)	FGR (*n* = 6)	Aged (*n* = 6)
Body (g)	660.3 ± 21.0	634.0 ± 36.1	0.65 ± 0.04*
Heart (g)	2.11 ± 0.11	2.11 ± 0.09	2.83 ± 0.30*
Kidney (g)	5.25 ± 0.20	4.27 ± 0.26*	4.82 ± 0.25
Rel. heart weight	0.32 ± 0.02	0.34 ± 0.01	0.44 ± 0.05*
Relative kidney weight	0.80 ± 0.03	0.65 ± 0.04*	0.75 ± 0.05

Values expressed as Mean ± SEM. Relative weight was defined as [organ weight] × [body weight^-1^] × 100. * *p* < 0.05, ** *p* < 0.01 vs control counterparts.

**Table 2:** Ex vivo vascular responses in carotid and femoral arteries.

**Carotid Artery**	**Control Fetus**	**FGR Fetus**	**Control Adult**	**FGR Adult**	**Control Aged**
Diameter (μm)	761 ± 16	675 ± 14*^#^	1323 ± 33*	1308 ± 43*	1250 ± 69*
KCl_Max_ (Nm^-2^)	1.41 ± 0.20	1.07 ± 0.10^#^	6.10 ± 1.26*	7.23 ± 1.06*	5.64 ± 1.10*
Ach_Max_ (%KCl)	72.9 ± 5.0	74.8 ± 7.8^#^	41.4 ± 5.4*	10.1 ± 11.0*^#^	15.6 ± 3.9*^#^
SNP_Max_ (%KCl)	94.6 ± 3.8	100.0 ± 4.7^#^	65.2 ± 5.3*	40.0 ± 5.2*^#^	22.7 ± 2.3*^#^
SNP pD_2_	6.99 ± 0.10	7.00 ± 0.10	6.98 ± 0.18	6.43 ± 0.20	6.25 ± 0.19*^#^
**Femoral Artery**	**Control Fetus**	**FGR Fetus**	**Control Adult**	**FGR Adult**	**Control Aged**
Diameter (μm)	497 ± 10	396 ± 17*^#^	648 ± 22*	631 ± 21*	697 ± 24*
KCl_Max_ (Nm^-2^)	2.57 ± 0.29	5.43 ± 0.72*^#^	8.86 ± 0.81*	6.91 ± 0.92*	6.97 ± 0.75*
Ach_Max_ (%KCl)	57.9 ± 6.2	56.1 ± 5.7^#^	87.6 ± 6.1*	44.4 ± 7.5^#^	51.7 ± 4.0^#^
SNP_Max_ (%KCl)	94.5 ± 3.2	95.4 ± 4.8	96.9 ± 4.4	89.7 ± 4.6	77.1 ± 3.9*
SNP pD_2_	7.78 ± 0.11	7.11 ± 0.10*	7.06 0.12*	6.44 ± 0.11*^#^	6.40 ± 0.11*^#^

Values expressed as Mean ± SEM. KCl_Max_, maximal KCl induced constriction; Ach_Max_, maximal acetylcholine-induced relaxation; SNP_Max_, maximal SNP-induced relaxation; SNP pD_2_, SNP potency (−LogEC50). *p < 0.05 vs. control fetuses, ^#^*p* < 0.05 vs control adults, ANOVA Tukey’s multiple comparison test.

## Abbreviations

Ach	Acetylcholine
CpG	Cytosine-guanine dinucleotide
CVD	Cardiovascular Diseases
EVA	Early Vascular Aging
EDR	Endothelial Relaxation
FGR	Fetal Growth Restriction
HUAEC	Human Umbilical Artery Endothelial Cells
KCl	Potassium Chloride
LINE-1	Long interspersed nuclear element-1
NO	Nitric Oxide
SNP	Sodium Nitroprusside
